# Impact of sodium caprate dosed as a mini-tablet or suspension on insulin delivery and mucosa histomorphology

**DOI:** 10.1007/s13346-025-01977-8

**Published:** 2025-10-08

**Authors:** Freja Fredholt, Joanne Heade, Jukka Rantanen, Stine Rønholt, Hanne Mørck Nielsen

**Affiliations:** 1 Department of Pharmacy, Faculty of Health and Medical Sciences, University of Copenhagen, Copenhagen, Denmark; 2 Center for Biopharmaceuticals and Biobarriers in Drug Delivery (BioDelivery), Faculty of Health and Medical Sciences, University of Copenhagen, Copenhagen, Denmark; 3 LEO Foundation Center for Cutaneous Drug Delivery, Faculty of Health and Medical Sciences, University of Copenhagen, Copenhagen, Denmark

**Keywords:** Permeation enhancer, C10, Mini-tablet, Solid dosage form, Preclinical testing, Compatibility assessment

## Abstract

Oral administration of solid dosage forms for delivery of therapeutic peptides is highly desired. Preclinical investigations on co-administration with permeation enhancers (PEs) to enable sufficient oral bioavailabilities are, however, predominantly done using liquid formulations despite the commercial end-goal being a solid dosage form. Given the amounts needed of PE, this will typically result in a compacted tablet with high amounts of the PE of choice. The aim of this study was to compare the pharmacokinetics (PK) and pharmacodynamics (PD) of insulin after co-formulation with a fixed dose of sodium caprate (C10) in solid dosage forms versus liquid dosage forms. PK/PD parameters in rats were evaluated after dosing mini-tablets and liquid formulations with different amounts of insulin and 26 mg/kg C10 after intestinal administration. Absorption of insulin was dose-dependent in the presence of the PE for both types of dosage forms, which was also reflected in the blood glucose levels. A significant absorption enhancing effect of C10 was found when dosing a 75 IU/kg insulin mini-tablet, resulting in a 26-fold increase in bioavailability. The effect of C10 on the rat intestinal tissue was investigated by histomorphological assessment evaluating erosion and villi height. Effects caused by the C10 mini-tablets and the liquid formulations were similar and shown to be transient. Overall, the findings in this study suggest that mini-tablets can be used to assess peptide bioavailability and the effect of PEs in rats as a preclinical model, and PK data may be nominally different from those obtained with liquid formulations.

**Graphical abstract:**

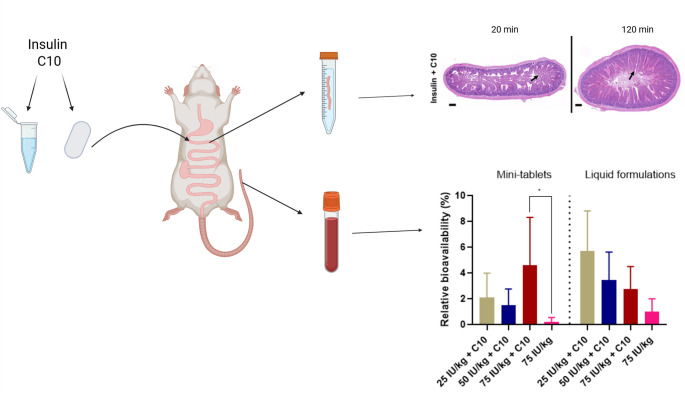

**Supplementary Information:**

The online version contains supplementary material available at 10.1007/s13346-025-01977-8.

## Introduction

Peptide and protein therapeutics are increasingly used for treatment of chronic and acute diseases due to their high potency and specificity [1, 2]. Sufficient systemic bioavailability upon oral administration of peptides and proteins is, however, challenged by their physicochemical properties, resulting in low stability in the gastro-intestinal (GI) fluids and poor permeation across the intestinal mucosa. Administration by injection is, therefore, still the most employed method of administration for peptides and proteins, despite the poor patient compliance associated with repeated injections [3, 4]. Research suggests different approaches to improve both stability and permeation to achieve sufficient absorption of the fully functional therapeutic across the intestinal mucosa to the systemic circulation. A useful strategy is co-administration or co-formulation of functional excipients, such as permeation enhancers (PEs), with peptide and protein drugs [4, 5]. However, only a few such formulations have reached the market [4], including the peptide semaglutide formulated with the PE sodium N-(8-[2-hydroxybenzoyl]amino) caprylate (SNAC) as a tablet (Rybelsus^®^) used to improve glycemic control in adults with diabetes mellitus type 2. Rybelsus^®^ contains 300 mg SNAC and only between 3 and 14 mg semaglutide resulting in an oral bioavailability of 0.4-1% in humans [6–8]. Another well-studied PE is C10, a sodium salt of the medium-chain fatty acid (MCFA) decanoic acid, which is believed to increase both the transcellular and paracellular permeation of co-formulated peptides and proteins [9–11]. C10 has been evaluated in clinical trials formulated as tablets in combination with either insulin or antisense oligonucleotides [12–14]. Further, studies have shown that co-administering C10 in liquid formulations can increase the intestinal absorption of peptides and of the macromolecule, fluorescein isothiocyanate-labelled dextran 4,000 Da (FD4) in rats following intra-intestinal administration [15–17]. This PE has also been shown to enhance the glucose-dependent insulinotropic polypeptide (GIP) and glucagon-like peptide-1 (GIP/GLP-1) dual agonist peptide (LY) and the GLP1 receptor agonist drug candidate MEDI7219 absorption in mini-pigs when co-formulated into enteric-coated capsules administered into the small intestine [9, 18]. Furthermore, Berg et al. reported increased absorption after intestinal administration of the GLP-1 receptor agonist MEDI7219 in mini-pigs, when the drug was co-formulated with C10 into mini-tablets, which were subsequently loaded into an intestinal administration device [18].

 In vivo evaluations of PEs for oral delivery of peptide drugs are most often done using liquid formulations, despite the end-goal being oral administration of a solid dosage form [19]. In vivo studies evaluating absorption of drugs, including biopharmaceuticals, are frequently (~ 80%) performed in rats dosed by oral gavage [19], and testing solid dosage forms such as tablets and capsules can be rather challenging. Indeed, studies investigating gastric capsule retention following oral gavage to rats, show that the dosage form does not consistently reach the intestinal lumen, thereby potentially affect the conclusions [20–22]. Administration by intestinal instillation/injection is a way to circumvent such problems with retention in the stomach but cannot be done in non-anesthetized animals. Bypassing the stomach gives the added benefit of avoiding the degradation of the peptide drug in the harsh gastric environment, and the approach allows for exclusive examination of intestinal absorption. A solid dosage form must disintegrate in the GI fluids to ensure the release and dissolution of both the active pharmaceutical ingredient (API) and the functional excipients, such as PEs. Disintegration of a compacted tablet is a complex process involving a series of physical phenomena, beginning with liquid penetration (wicking) into the pores of the powder compact, which is often the rate-limiting step. Once liquid penetrates into the compact, several mechanisms can be initiated, including swelling (omnidirectional expansion of particles), straining (unidirectional expansion of particles), and the dissolution of soluble excipients from the pore walls. These mechanisms, either individually or in combination, disrupt particle-particle bonds, ultimately leading to disintegration [23–25]. Following disintegration, both the API and the PE must dissolve to enable permeation enhancement and facilitate API absorption. It is thus crucial that a sufficient volume of fluid is present to dissolve the often high doses of PE. In the human small intestine, the total fluid volume is approximately 105 mL [26], whereas in the rat small intestine, it is lower (~ 1.9 mL) [27], which may present challenges for the disintegration process. Also, other conditions in the rat model such as the continuous bile secretion into the intestine may influence the outcome [16].

The aim of the study was to compare the efficacy and compatibility of compacted mini-tablets with insulin and a high dose of C10 to the corresponding doses administered as a liquid. Insulin was used as a model peptide allowing for a rapid pharmacological response and reliable detection of in vivo effects after intra-intestinal dosing of liquid formulations containing PEs [28]. The mini-tablets were produced by direct compression and administered to the rat small intestine via intestinal instillation. We evaluated the pharmacokinetics (PK) and pharmacodynamics (PD) readouts and performed histomorphological assessments of the mucosal tissue after exposure to the formulation for 20 and 120 min. Different doses of insulin were evaluated (25, 50, and 75 international units (IU)/kg) while maintaining the C10 dose (26 mg/kg).

## Materials and methods

### Materials

Human recombinant insulin (5808 g/mol, >27.5 IU/mg, < 1% zink, amorphous powder), barium sulfate (BaSO_4_, 99.99%, crystalline powder) was obtained from Merck (Darmstadt, Germany). Sodium decanoate (> 99.0%, crystalline powder) was purchased from TCI Europe (Zwijndrecht, Belgium). Crospovidone (Kollidon^®^ CL-F) and copovidone (Kollidon^®^ VA64 Fine) was kindly donated by BASF (Ludwigshafen, Germany). Magnesium stearate (Ph. Eur.) was from Alfa Aesar Pharma (Loughborough, UK). Microcrystalline cellulose (Avicel PH-101) and trifluoroacetic acid (TFA) was purchased from Merck (Darmstadt, Germany). Monohydrate lactose (Supertab^®^ 11Sd) was purchased from DFE Pharma (Goch, Germany). Hank’s balanced salt solution (HBSS), bovine serum albumin (≥ 98%) (BSA), sodium phosphate dibasic heptahydrate (98.0-102.0%), sodium phosphate monobasic monohydrate (≥ 98%), formalin solution, neutral buffered 10%, Mayer’s hematoxylin solution, and eosin Y solution (alcoholic) were obtained from Merck (Darmstadt, Germany). 4-(2-hydroxyethyl)-1-piperazine-1-ethanesulfonic acid (HEPES) was purchased from PanReac AppliChem (Darmstadt, Germany). Hydrochloric acid (HCl) 5.0 N, sodium hydroxide (NaOH) 5.0 N in aqueous solution and sodium chloride (NaCl) (Ph. Eur.), Supelco^®^ was purchased from VWR Chemicals BDH International (Leicestershire, UK). Midazolam 5 mg/mL was manufactured by Hameln Pharma (Gloucester, UK). Hypnorm containing fentanyl citrate 31.5 µg/mL and fluanisone 1 mg/mL was produced by Skanderborg Pharmacy (Skanderborg, Denmark). Euthasol^®^ vet. containing pentobarbital 400 mg/mL was produced by Dechra Veterinary Products A/S (Uldum, Denmark). Ultrapure water was collected in-house (18.2 MΩ × cm by a PURELAB flex 4 system (ELGA, LabWater, High Wycombe, UK).

### Preparation and characterization of mini-tablets

#### Preparation of mini-tablets

Mini-tablets for the PK/PD studies were composed of amorphous insulin, crystalline BaSO_4_, crystalline C10, and standard tablet excipients (Tables 1 and 2). In all cases, bulk volumes of the weighed ingredients were mixed manually in a mortar followed by weighing out the powder for each individual mini-tablet before compaction. The powder was directly compacted into elongated tablets (6 × 2 mm) on a HB10 compaction simulator (Huxley Bertram, Cambridge, UK) with a compaction force of 3.5 kN. All mini-tablets were stored at room temperature in a sealed container until use, for a maximum of 4 days prior to the in vivo studies and maximum 4 weeks before the in vitro experiments. The compositions of the mini-tablets with BaSO_4_ prepared for transit studies can be seen in Table 1 and the compositions of the mini-tablets prepared for PK/PD studies are shown in Table 2. One unit of insulin corresponds to 0.0347 mg of insulin.

**Table 1:** Composition (%, w/w) of mini-tablets for transit studies

Composition (%, w/w)	Mini-tablet BaSO_4_ + C10	Mini-tablet BaSO_4_
BaSO_4_	26	26
C10	35.5	-
Kollidon^®^ CL-F	3	3
Kollidon^®^ VA64 Fine	2.5	2.5
Magnesium stearate	1	1
Avicel PH-101:SuperTab^®^ 11SD (50:50)	32	67.5

**Table 2:** Composition (%, w/w) of mini-tablets containing varying doses of insulin and the PE C10

Composition of mini-tablet (%, w/w)	25 IU/kg insulin + C10	50 IU/kg insulin + C10	75 IU/kg insulin + C10	75 IU/kg	C10
Insulin	1.2	2.5	3.7	3.7	-
C10	35.5	35.5	35.5	-	35.5
Kollidon^®^ CL-F	3	3	3	3	3
Kollidon^®^ VA64 Fine	2.5	2.5	2.5	2.5	2.5
Magnesium stearate	1	1	1	1	1
Avicel PH-101:SuperTab^®^ 11SD (50:50)	56.8	55.5	54.3	89.8	58

#### Tensile strength

The total height and the wall height of the mini-tablets were carefully measured using a caliper. Afterwards, the break force was measured using a tablet hardness tester (MT-2 – Sotax, Aesch, Switzerland). Equation 1 was used to calculate the tensile strength [29].

1 $$\begin{array}{l}\:Tensile\:strength\:\left(TS\right)\\=\:\frac{2}{3}\left(\frac{10P}{\pi\:{D}^{2}\:\left(2.84\frac{t}{D}-0.216\frac{t}{W}+3.15\frac{w}{D}+0.01\right)}\right)\end{array}$$

Where TS is the tensile strength [Pa], P is the breaking force [N], D is the length of the short axis [m], t is the overall height of the mini-tablet [m] and W is the wall height of the mini-tablet [m].

#### Disintegration of mini-tablets

Disintegration time was determined with an in-house set-up by placing a mini-tablet in a 24-well plate (Corning, Costar Transwell^®^, Thermo Fisher Scientific, Roskilde, Denmark) containing 0.4 mL of 0.1 M phosphate-buffered saline (PBS), prepared using sodium phosphate dibasic heptahydrate and sodium phosphate monobasic monohydrate in purified water. The pH was adjusted to 6.5, and the osmolality was regulated to 310 mOsm using NaCl. The plate was placed in a heating cabinet at 37 °C with shaking at 125 rpm. Disintegration times were determined by visual inspection (*n* = 4).

#### Release of insulin from mini-tablets

Examination of the insulin release was done by placing a mini-tablet in a Protein LoBind^®^ Eppendorf^®^ tube (Eppendorf^®^, Hamburg, Germany) with 0.4 mL of 0.1 M phosphate buffered saline (PBS) prepared as described above. The tubes were placed in a PCMT Thermoshaker (Grant Instruments, Royston, UK) at 37 ˚C and 250 rpm. Samples of 25 µL were taken at 0, 20, 40, 60, 90, 120, 180, and 360 min and the removed volume was replaced with PBS at each sampling time. The samples were appropriately diluted, and insulin was quantified by reversed-phase high performance liquid chromatography (RP-HPLC) using an Agilent 1200 system (Agilent, Santa Clara, CA, USA) equipped with a Kinetex^®^ XB-C18 column (2.6 μm, 100 Å, 50 × 2.1 mm; Torrance, CA, USA) at 40 ˚C. The mobile phase A consisted of 95% (v/v) ultrapure water containing 0.1% (v/v) TFA mixed with 5% (v/v) acetonitrile and the mobile phase B consisted of 95% (v/v) acetonitrile mixed with 5% (v/v) water containing 0.1% (v/v) TFA. A linear gradient elution from 20% to 70% mobile phase B over 12 min was used with a flow rate of 0.7 mL/min and an injection volume of 30 µL with a column temperature of 40 ˚C. The absorbance was measured using a photodiode Array (PDA) detector at 218 nm. The limit of detection (LOQ) for insulin was determined to be ≤ 20 µg/mL.

### Preparation of insulin-C10 liquid formulations

Isohydric and isoosmolar liquid formulations with identical doses of insulin and C10 as in the mini-tablets were prepared anticipating an average rat weight of 300 mg and an intended dosed volume of 0.4 mL for dosing of 25, 50, or 75 IU/kg insulin with or without 26 mg/kg C10 per rat. The 0.1 M PBS was prepared as mentioned above in the Disintegration of mini-tablets section. The pH was adjusted to either 7.4 or 6.5 and osmolality was adjusted with NaCl to 399–412 mOsm/kg and 308–320 mOsm/kg, respectively. A C10 stock was prepared by suspending C10 in 0.1 M PBS pH 6.5 to give a concentration of 80 mg/mL. An insulin stock solution was prepared by dissolving insulin in 0.01 M HCl to give concentrations of 2.333 mg/mL, 4.665 mg/mL, and 7 mg/mL. Insulin-C10 suspensions with 25, 50, and 75 IU/kg insulin doses for a 300 g rat were prepared by mixing 0.195 mL of the C10 stock with 0.372 mL 0.1 M PBS adjusted to pH 6.5, and 0.233 mL of each of the insulin stocks with 0.1 M PBS. The C10 suspension was prepared by mixing 0.195 mL C10 stock with 0.372 mL 0.1 M PBS pH 6.5 and 0.233 mL 0.01 M HCl. Insulin solution without C10 was prepared by mixing 0.567 mL of 0.1 M PBS pH 7.4 and 0.233 mL of 7 mg/mLinsulin stock solution. Osmolality and pH were measured for all the final formulations.

### In vivo studies

All animal studies were carried out in sedated male Sprague Dawley (SpD) rats under the license number 2021-15-0201-00856 approved by the Danish Animal Experiments 31 Inspectorate and in compliance with Danish laws regulating experiments on animals and EU directive 2010/63/EU. All rats were housed together in reversed day/night rhythm (12/12 h) in groups of minimum 2 rats and maximum 6 rats. Standard laboratory chow and water were available ad libitum and cage enrichment were provided. Fasted rats had continuous access to water during the entire period. The rats were sedated with subcutaneous (s.c.) injection into the loose skin over the rat neck with an initial dose of 2 mL/kg of a solution containing 78.75 µg/mL fentanyl citrate, 2.5 mg/mL fluanisone, and 1.25 mg/mL midazolam in purified water. Maintenance doses of 1 mL/kg were injected s.c. every 30 min for 1 h and then 0.5 mL/kg every 30 min for the remaining surgery time.

#### Transit of BaSO_4_ mini-tablets after intestinal instillation

The animal study was performed in sedated fasted (18 h) SpD rats (304 ± 13 g (mean ± SD, *N* = 6). A small incision was made in the small intestine, on average 5.1 ± 0.9 cm (mean ± SD) from the stomach, and a mini-tablet containing 26% (w/w) BaSO_4_ with or without C10 was placed in the intestine at the incision point and gently moved 2 cm down the intestine using a rounded metal rod to ensure the mini-tablet was not in direct contact with the incision wound. The incisions were closed with glue (Loctite Super Glue Power Flex Control, Loctite, Düsseldorf, Germany). At 5 and 20 min after administration, the rats were euthanized using intracardiac injection with pentobarbital (100 mg/kg). The GI tracts were immediately removed, placed in petri dishes and X-ray images were captured maximum 5 min after euthanization using an IVIS Lumina XR (Xenogen Corporation-Caliper, Alamanda, CA, USA). X-rays were generated using a voltage of 28 kV and a current of 100 µA. After obtaining the X-ray images, the GI tracts were carefully removed, opened, and the mini-tablets position from the stomach were measured and disintegration was evaluated visually.

#### PK/PD after intestinal instillation of insulin-C10 mini-tablets

Intestinal instillation was carried out in sedated fasted (15 h) SpD rats (307 ± 15 g (mean ± SD), *N* = 28). A small incision was made in the upper small intestine, on average 4.1 ± 0.8 cm (mean ± SD) downwards from the stomach, and a mini-tablet was placed in the intestine at the incision point and moved 2 cm down the intestine using a rounded metal rod to avoid interaction with the site of incision. The mini-tablets were assigned to the rats considering the weight of both the mini-tablets and the rats, resulting in doses of 26 mg/kg C10 and either 25, 50, and 75 IU/kg insulin as accurately as possible. As controls, six rats were injected s.c. with 1 IU/kg insulin dissolved in PBS. For all rats, 200 µL blood samples were drawn from the tail vein at 0, 15, 30, 60, 90, 120, 180, 240, and 360 min into a Microvette^®^ 200 K3S (Sarstedt, Nümbrecht, Germany) followed by isolation of the blood plasma by centrifugation for 10 min at 10,000 × g at 4 °C using a Sorvall™ Legend™ Micro 17r microcentrifuge (Thermo Fischer, Waltham, MA, USA). The plasma samples were stored at -20 °C until quantification of insulin was carried out by an enzyme-linked immunosorbent assay (ELISA) as described by the manufacturer (Crystal Chem, Zaandam, Netherlands). Briefly, samples were diluted to be within the range of the standard curve (0.078–5.266 ng/mL) and standard samples were prepared in 10 mM HEPES in HBSS supplemented with 0.05% (w/v) BSA. Blood glucose was determined immediately after the blood sampling by using a Contour^®^ XT meter (Ascensia Diabetes Care, Basel, Switzerland). All rats were sacrificed at the end of the experiment, i.e. immediately after collecting the 240 min sample, by euthanization with 90.5% carbon dioxide in oxygen. Rats that expired during the study were excluded from data analysis. The relative bioavailability (BA) was calculated by Eq. (2),

2 $$\:Relative\:BA=\:{\left(AUC/dose\right)}_{i.i.\:}/{\left(AUC/dose\right)}_{s.c.}\times\:100\:\%$$

where AUC is the area under the curve for plasma insulin following intra-intestinal (i.i.) and subcutaneous (s.c.) administration.

The relative biopotency (BP) was calculated by Eq. (3),

3 $$\:Relative\:BP=\:{\left(AOC/dose\right)}_{i.i.\:}/{\left(AOC/dose\right)}_{s.c.}\times\:100\:\%$$

where AOC is the area over the curve for blood glucose following intra-intestinal (i.i.) and subcutaneous (s.c.) administration.

#### PK/PD after intestinal injection of insulin-C10 liquid formulations

Intestinal injection was carried out in sedated fasted (15 h) SpD rats (297 ± 9 g (mean ± SD, *N* = 25). A silicone catheter (0.96 (outer diameter) × 0.58 (inner diameter) × 380 (length) mm) was carefully inserted in the upper small intestine, on average 6.4 ± 1.0 cm (mean ± SD) downwards from the stomach and moved 2 cm further into the intestine. Corrected for the precise weight of the rat, a volume between 0.37 and 0.42 mL of test sample was administered through the catheter, resulting in the administration of 26 mg/kg C10 (~ 100 mM) and either 0, 25, 50, or 75 IU/kg insulin. Insulin was dissolved in the liquid, whereas C10 was suspended. Following administration, the catheter was flushed with 100 µL air (corresponding to the dead volume of the catheter) to ensure complete administration of the sample. Blood samples were drawn and analyzed as described in the PK/PD after intestinal instillation of insulin-C10 mini-tablets section. At the end of the experiment, all rats were sacrificed as described above and rats that expired during the study were excluded from the data analysis.

#### Histomorphological assessment of intestinal tissue

Hematoxylin and eosin (H&E) staining was conducted to investigate any effects on the rat intestinal tissue. Intestinal instillations and intestinal injections were performed as described above. The rats were fasted (18 h) and had an average weight of 302 ± 14 g (mean ± SD, *N* = 18) and 283 ± 9 g (mean ± SD, *N* = 19) for instillation and injection studies, respectively. By the same process as described above, a small incision was made in the upper small intestine, in average 4.5 ± 0.9 cm (mean ± SD) downwards from the stomach for the mini-tablets and in average 5.7 ± 1.3 cm (mean ± SD) downwards from the stomach for the liquid formulations. Mini-tablets or liquid formulations with 26 mg/kg C10 and either 0 or 75 IU/kg insulin or only 75 IU/kg insulin were administered. As control, rats (317 ± 5 g (mean ± SD, *n* = 5)) were injected s.c. with 1 IU/kg insulin. At 20–120 min post administration, the rats were euthanized by intracardiac injection of pentobarbital (100 mg/kg). The small intestine was carefully isolated 6 cm downwards from the incision point and preserved in 10% (v/v) neutral buffered formalin for 24 h. Following this, tissue samples were processed using a Leica ASP300S fully enclosed tissue processor (Leica Biosystems, Wetzlar, Germany), embedded in paraffin using a Tissue-Tek TEC 5 tissue embedding console system (Sakura Finetek Europe, Alphen aan den Rijn, Netherlands), and 5 μm sections were cut using a Thermo Scientific HM 355 S automatic microtome package and mounted on slides (StarFrost^®^ adhesive slides, Houisen Laboratorieudstyr A/S, Skanderborg, Denmark). The tissue samples were stained with H&E and scanned using a NanoZoomer-XR Digital slide scanner C12000-01 (Hamamatsu Photonics, Shizuoka, Japan) in 40× mode, which yields a spatial resolution of 0.23 μm/pixel. Tissue sections were blinded, and the effects of mini-tablets and liquids were evaluated by assessing each section for erosion (epithelial or lamina propria) and morphological changes, as described by Raptis et al. [30]. Briefly, the baseline morphology was established by measuring the height of each villus present in untreated intestinal samples harvested at 120 min (s.c. control animals, *N* = 3, *n* = 1, 39 ± 1 villi per N (mean ± SD), 117 villi total). These measurements were carried out in the Nanozoomer Digital Pathology viewer (NDP.view 2.7.25, Hamamatsu Photonics) at various on-screen magnifications (up to the optical equivalent of 40×) as required for accurate assessment, typically in the 10–20× range. Data were analyzed in Python using K-means clustering. Two clusters were identified, with a cut-off point of 475 μm (Figure S1). Using this cut-off, morphological changes were assessed by classifying each villus according to its height: “Tall” (≥ 475 μm) or “Short” (< 475 μm). Data were collected for *N* = 2–5, *n* = 1 (32.95 ± 6.98 villi per N (mean ± SD), 1450 villi total).

### Data analysis

Data analysis was performed with Microsoft Office Excel Professional Plus 2016 (Microsoft, Houston, TX, USA) and GraphPad Prism 9 (GraphPad Software, San Diego, CA, USA). Data are presented as mean and standard derivation (SD) or mean and standard error (SEM). N represents biological replicates whereas n represents technical replicates. Statistical analysis was performed with GraphPad Prism 9 using two-way analysis of variance (ANOVA; one-way for erosion data and two-way for morphology data) with Turkey’s multiple comparison.

## Results

### Physical characteristics of the mini-tablets

**Fig. 1:**
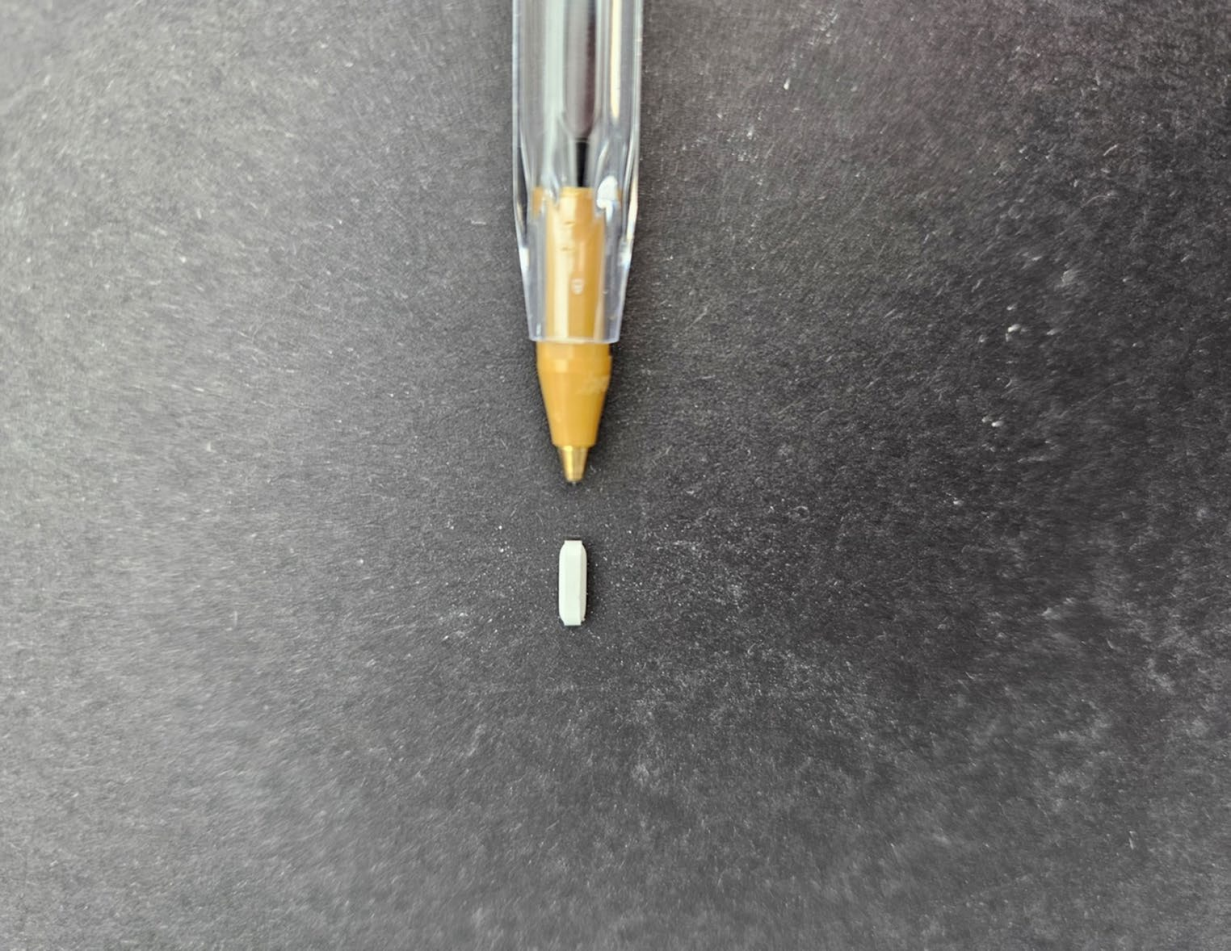
Appearance of produced mini-tablet

All the mini-tablets (Fig. 1) had a fixed length of 6 mm and a fixed width of 2 mm and weighed around 22 mg. The measured heights were approximately 2 mm, although the tablets without C10 had a slightly lower value (*p* < 0.0001) (Table 3). For characterization, the tensile strength was investigated by measuring the break force and no significant differences were observed between the calculated tensile strengths for the mini-tablets containing C10 (Table 3). However, the mean calculated tensile strength for the mini-tablets with 75 IU/kg insulin without any C10 was significantly higher (*p* < 0.0001) than for the mini-tablets with C10, which was also reflected in a longer disintegration time when evaluated in 0.4 mL PBS at 37 ˚C. For all the mini-tablets containing C10, the disintegration time was longer than 10 min and significantly higher (*p* < 0.0001) than for the mini-tablets without C10. Thus, incorporating 35.5% (w/w) of crystalline C10 into mini-tablets together with insulin and other standard excipients was indeed possible.

**Table 3:** Tensile strength, height, weight, and disintegration time of the mini-tablets with insulin and C10 (mean ± SD, *n* = 6 for tensile strength and height and *n* = 12–24 for weight and *n* = 4 for disintegration time)

Mini-tablet	Tensile strength (MPa)	Height (mm)	Mass (mg)	Disintegration time (min: s)
25 IU/kg insulin + C10	1.9 ± 0.1	1.96 ± 0.01	22.1 ± 0.2	11:06 ± 00:19
50 IU/kg insulin + C10	2.0 ± 0.1	1.97 ± 0.01	22.2 ± 0.2	11:03 ± 00:18
75 IU/kg insulin + C10	2.2 ± 0.2	1.97 ± 0.03	22.2 ± 0.2	11:02 ± 00:16
75 IU/kg insulin	5.0 ± 0.2	1.85 ± 0.01	22.3 ± 0.2	4:46 ± 00:50
C10 alone	1.8 ± 0.1	1.94 ± 0.01	22.3 ± 0.2	10:10 ± 00:14

#### In vitro insulin release from mini-tablets

**Fig. 2:**
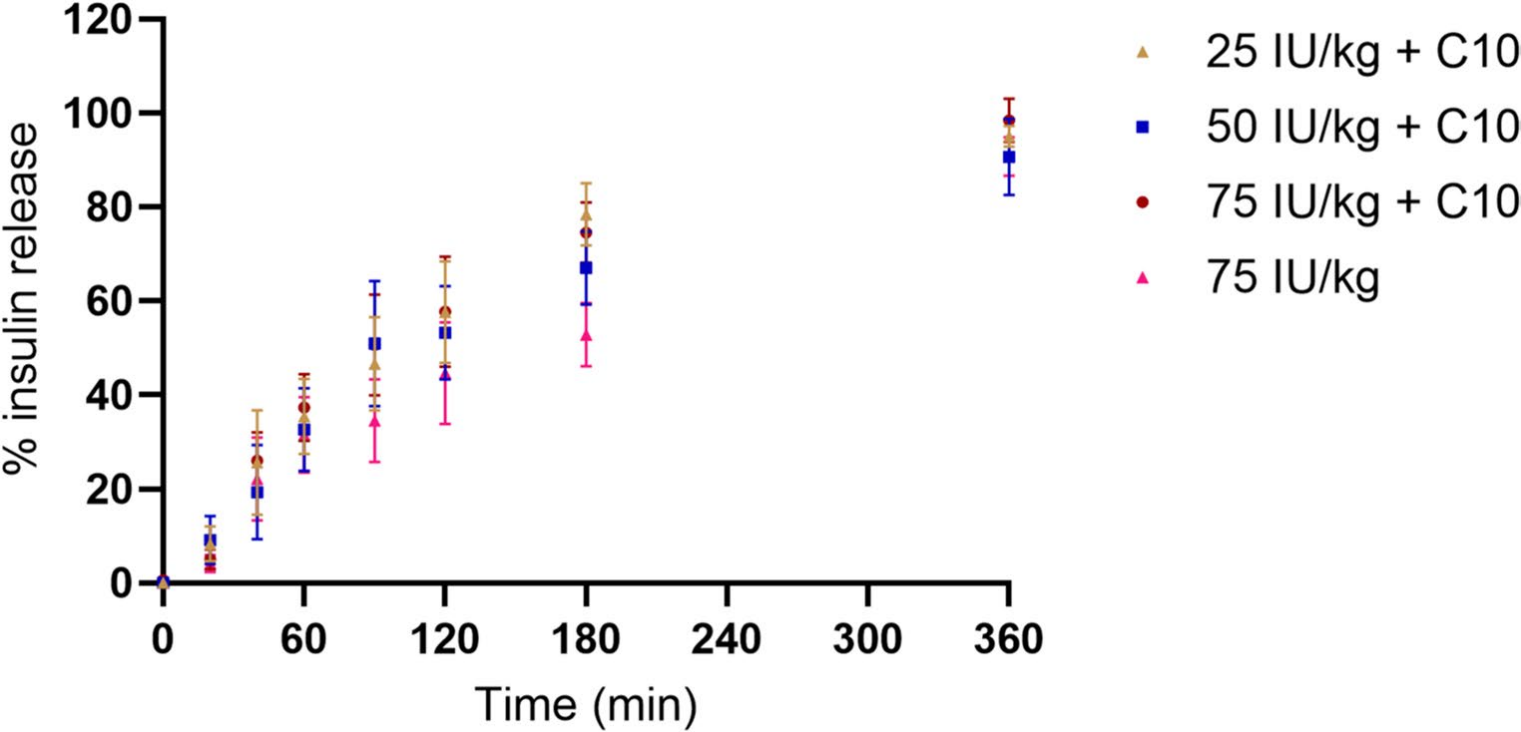
Percentage release of insulin over time from mini-tablets with 25 IU/kg insulin + C10, 50 IU/kg insulin + C10, 75 IU/kg insulin + C10 and 75 IU/kg insulin in 0.1 M PBS buffer pH 6.5 (mean ± SD, *n* = 5–6)

The release of insulin from the mini-tablets (Fig. 2) was investigated in 0.4 mL PBS in Eppendorf^®^ tubes in mini-tablets with C10 and either 25, 50, or 75 IU/kg insulin as well as for the mini-tablets with 75 IU/kg insulin and no C10. At the first sample point, i.e. 20 min, all mini-tablets were fully disintegrated. The release profiles represented relative to the total content of insulin for all four formulations show a similar gradual release of insulin over 360 min, thus neither the amount of insulin or the presence of C10 changed the release kinetics. At 360 min, 95.0 ± 2.2% (mean ± SD) was released for the 25 IU/kg insulin + C10, whereas it was 93.7 ± 3.7%, 98.5 ± 4.6 and 90.8 ± 4.1 (means ± SDs) for 50 IU/kg insulin + C10, 75 IU/kg insulin + C10, and 75 IU/kg insulin, respectively.

### Mini-tablets disintegrate after instillation in rat small intestine

**Fig. 3:**
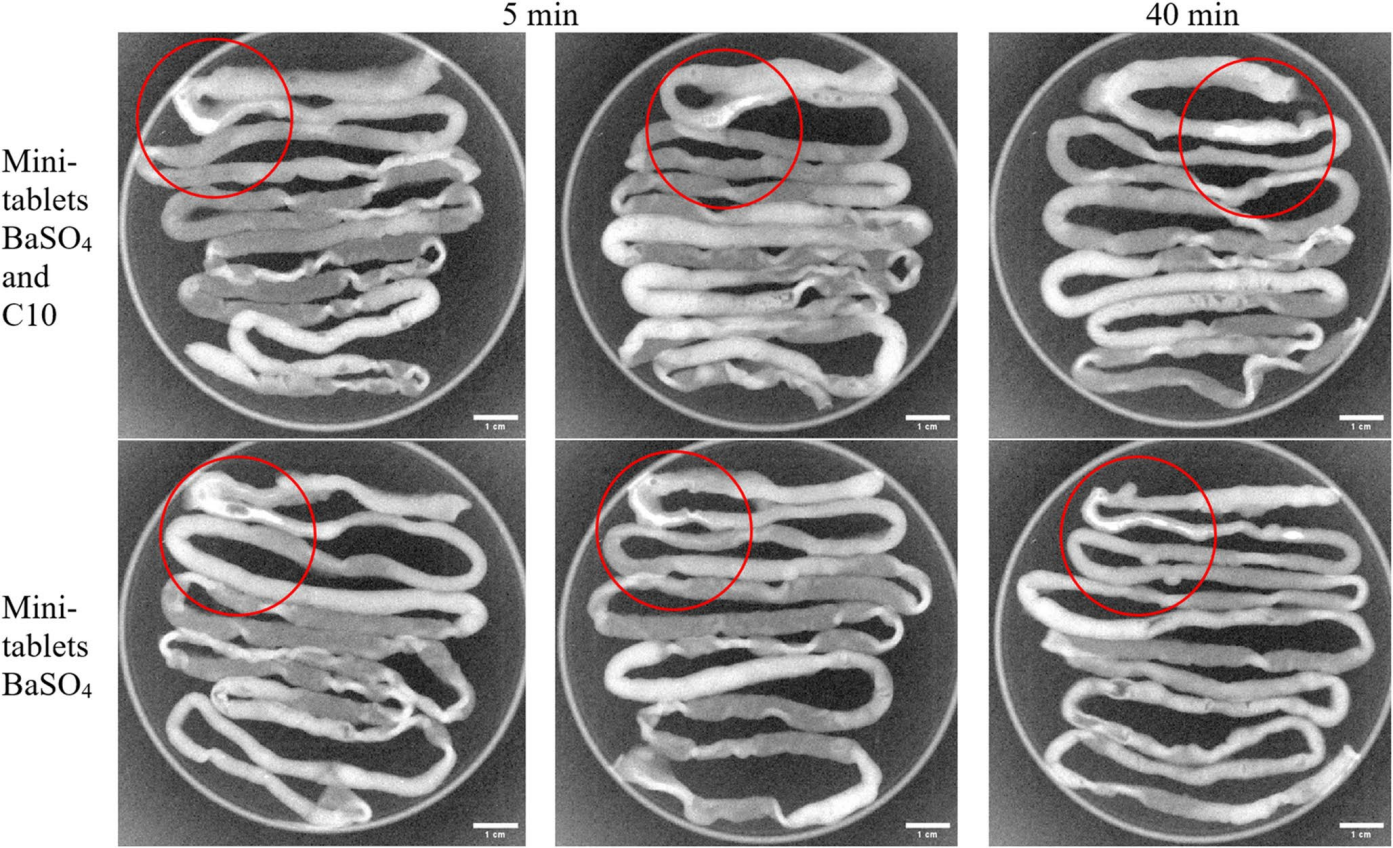
X-ray images of intestines for evaluating disintegration and transit of mini-tablets with and without C10 (35.5%) at 5 min (*N* = 4) and 40 min (*N* = 2) after intestinal instillation in rats. The red circles indicate the mini-tablet position. Scalebar 1 cm

Intestinal instillation of mini-tablets containing BaSO_4_ was conducted to investigate the transit and disintegration behavior. The inclusion of BaSO_4_ enabled detection of the mini-tablet using X-ray imaging. C10 was included in half (*n* = 3) of the mini-tablets to investigate if the excipient influenced the transit and disintegration behavior. These mini-tablets were compacted using the same punch as the mini-tablets with insulin and thus had a length of 6 mm and a diameter of 2 mm. After compaction, the mini-tablets with C10 weighed 24.5 ± 0.14 mg with a height of 1.84 ± 0.02 mm (mean ± SD). The mini-tablets without C10 weighed 24.89 ± 0.12 mg and had a height of 1.74 ± 0.01 mm (mean ± SD). Figure 3 displays the location of the mini-tablets at 5 and 40 min after instillation. Both images at 5 and at 40 min show dilution of the BaSO_4_ signal along the intestinal lumen, indicating disintegration of the mini-tablets in the intestinal fluid, regardless of whether the tablets contained C10 or not. All of the tablets moved less than 2 cm from the instillation site over the course of this 40-min study, indicating that the mini-tablets had limited movement after instillation in the intestines, likely due to decreased intestinal motility as a result of the sedative effect from the use of anesthetics [19]. The same is expected for mini-tablets administered in the PK/PD studies, and the in vitro release study with mini-tablets containing insulin and the same amount of C10 showed that they disintegrated within 10 min in buffer (see In vitro insulin release from mini-tablets section).

### C10 in mini-tablets and liquid formulations increased insulin bioavailability to similar levels

**Fig. 4:**
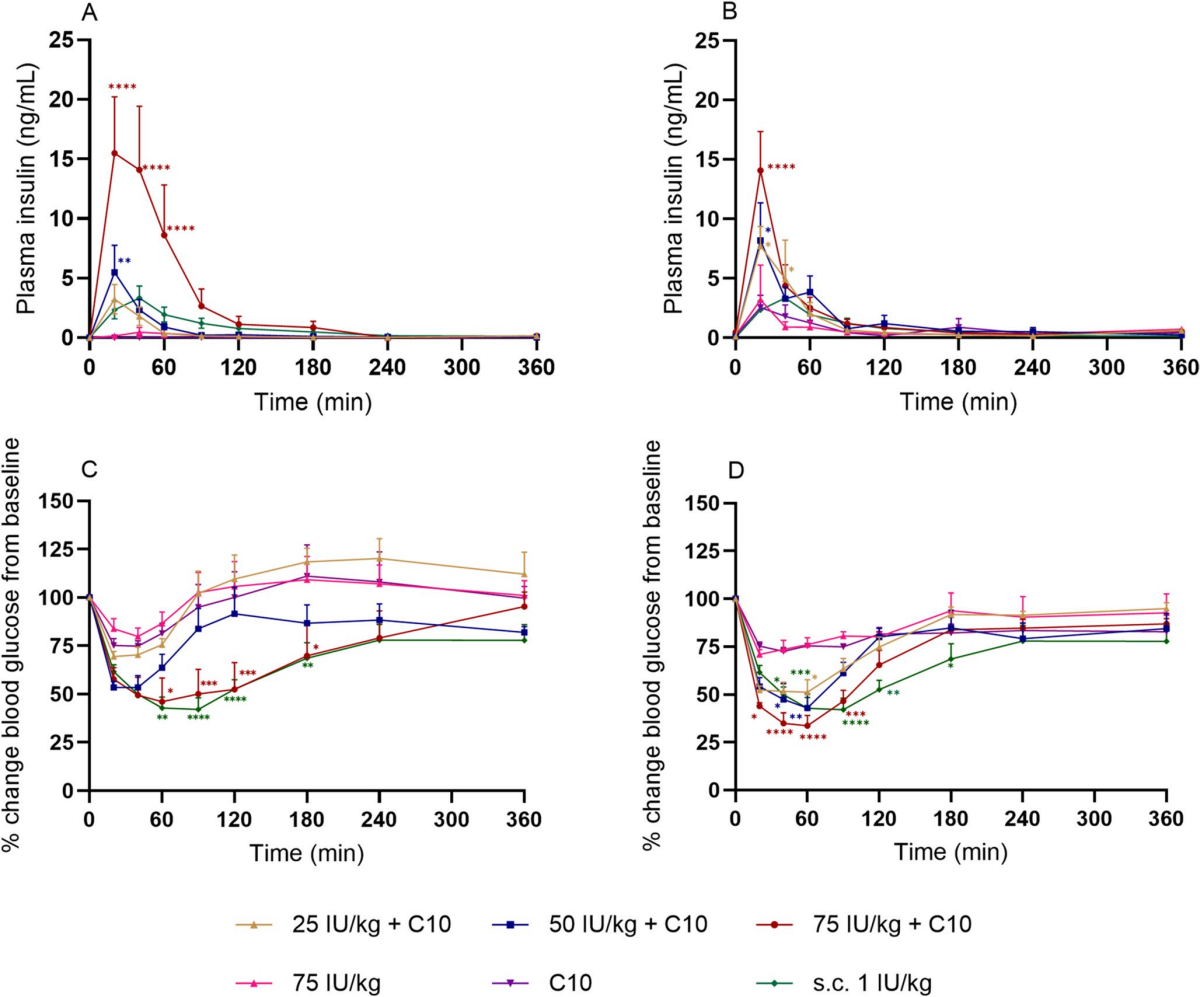
Plasma insulin (ng/mL) over time following intestinal administration to rats of mini-tablets (**A**) and liquid formulations (**B**) with insulin (0, 25, 50, and 75 IU/kg) and permeation enhancer C10. The relative blood glucose changes from baseline following intestinal administration of mini-tablets (**C**) and liquid formulations (**D**) with insulin (0, 25, 50, and 75 IU/kg) and the permeation enhancer C10. As a control, subcutaneous administration (s.c.) of 1 IU/kg insulin was used. Mean ± SEM (*n* = 5–6). Significant differences are *: *p* < 0.05, **: *p* < 0.01, ***: *p* < 0.001, and ****: *p* < 0.0001 relative to data obtained after dosing the 75 IU/kg insulin mini-tablet

The absorption of insulin after administration of the mini-tablets was evaluated and showed that inclusion of the PE C10 (26 mg/kg) in the mini-tablets increased the absorption of insulin. As a result, 26-fold increases in C_max_ were found for the mini-tablets with C10 and 75 IU/kg (*p* = 0.0021) insulin, compared to the mini-tablet with 75 IU/kg insulin alone (Table 5). Figure 4A shows similar PK profiles for the mini-tablets containing C10 and insulin at different doses with a T_max_ at around 20 min. The higher the insulin amount in the formulation, the larger AUC values were obtained (Table 5) and for 75 IU/kg with C10, the calculated mean AUC was significantly higher than the mean AUC obtained without C10 (*p* = 0.0007). The relative bioavailability resulted in 0.18 ± 0.15% for the 75 IU/kg mini-tablets and displayed a significant increase to 4.61 ± 1.66% (*p* = 0.0287) when dosed in combination with C10 (Table 5). Comparing the performance of the mini-tablets to the liquids, insulin absorption was also assessed after intestinal injection of insulin-C10 liquid suspensions in rats. The liquids were adjusted in pH (~ 7.3) and osmolality (~ 310 mOsm/kg) to mimic what was previously measured in intestinal fluid of fasted SpD rats (Table 4) [31].

**Table 4:** pH and osmolality (mOsm/kg) of the liquid formulations with and without insulin and the PE C10. Mean ± SD (*n* = 5–6)

	25 IU/kg insulin + C10	50 IU/kg insulin + C10	75 IU/kg insulin + C10	75 IU/kg insulin	C10
pH	7.30 ± 0.03	7.29 ± 0.04	7.32 ± 0.04	7.27 ± 0.07	7.32 ± 0.07
Osmolality (mOsm)	312.5 ± 9.4	311.0 ± 4.3	313.4 ± 3.5	298.4 ± 2.2	315.4 ± 4.2

Like the observations for the mini-tablets, the inclusion of the PE C10 (26 mg/kg corresponding to 100 mM) in the liquids demonstrated a tendency to increase the absorption of insulin. This could be seen from the C_max_ where a 4-fold increase was found for the C10 formulations with 75 IU/kg (*p* = 0.0884) insulin, compared to the C_max_ obtained after dosing a liquid formulation with 75 IU/kg insulin alone (Table 5). T_max_ was reached at 20 min for all the liquid formulations containing insulin administered by intestinal injection compared to the s.c. administration of insulin where T_max_ was reached at 40 min (Fig. 4B). The AUC value increased slightly when more insulin was present in the formulation and for all formulations containing C10, the AUC values tended to be higher than after s.c. injection of insulin. The relative bioavailability of the 75 IU/Kg insulin solution was 1.00 ± 0.99%, and while it significantly increased to 5.71 ± 3.11% when dosing 25 IU/kg with C10 (*p* = 0.0245), it increased less (2.76 ± 1.75%, non-significant) when 75 IU/kg insulin was dosed in a C10 suspension.

### Formulations with C10 in both mini-tablets and liquids decreased blood glucose levels in rats

The blood glucose levels were monitored after administration of both mini-tablets and liquid formulations containing insulin (Fig. 4C, D). Significant decreases in initial blood glucose levels were observed between 60 and 180 min after administration for the mini-tablets with 75 IU/kg insulin and C10 and for s.c. injection compared to the blood glucose levels of mini-tablets with 75 IU/kg insulin without the PE. (Fig. 4C). A similar trend was observed for the liquid formulations where a significant decrease in initial blood glucose levels were observed in the time interval 20–180 min after administration with 75 IU/kg insulin + C10, 25 IU/kg + C10, 50 IU/kg + C10, and after s.c. injection compared to administration of a solution with 75 IU/kg insulin without the PE (Fig. 4D). Overall, a decrease in blood glucose was observed following the administration of all formulations. The slight decrease in blood glucose observed after administration of the mini-tablet and liquids with only C10 and no insulin displays the effect of anesthesia and stress of surgery. The mini-tablet formulations with 75 IU/kg insulin + C10 and the s.c. injected formulation resulted in a prolonged decrease in blood glucose levels compared to the other mini-tablet formulations with maximum effects at 60 min (46.0%) and 90 min (42.1%) for 75 IU/kg + C10, and s.c., respectively. For the liquid formulations with insulin and C10, both 75 IU/kg insulin + C10 and the s.c. formulation showed an extended decrease in blood glucose, compared to that observed with the other formulations, with maximum decreases reached at 60 min (33.7%) and 90 min (42.1%), respectively.

For all solid and liquid formulations, an increase in blood glucose levels towards the baseline was observed over the course of the experiment time. The biopotency values for the administered mini-tablets and liquids compared to the s.c. administered insulin (Table 5) show that the calculated biopotencies for insulin formulations including the PE were larger than the calculated biopotency obtained for insulin administered alone. However, a significant difference was only observed for the suspension with 75 IU/kg insulin + C10 compared to administration of 75 IU/kg insulin solution alone (*p* = 0.003).

**Table 5:** Pharmacokinetics and pharmacodynamics parameters AOC, BP, AUC, BA, C_max_, and T_max_ calculated following intestinal administration of mini-tablets and liquid formulation with insulin (0, 25, 50, and 75 IU/kg) and the permeation enhancer C10 to rats. As a control, subcutaneous administration of 1 IU/kg was used. Mean ± SEM (*N* = 5–7) except for T_max_, which is reported as mean ± range. Significant differences are *: *p* < 0.05, **: *p* < 0.01 relative to the formulation with 75 IU/kg insulin and no C10

Formulation	Area over curve (AOC) (min × mmol/L)	Biopotency versus s.c. (BP) (%)	Area under curve (AUC) (min × ng/mL)	Bioavailiability versus s.c. (BA) (%)	C_max_ (ng/mL)	T_max_ (min)
s.c. injection	665.64 ± 64.59		285.31 ± 61.66		3.66 ± 0.95	40 (20/180)
**Mini-tablet**						
25 IU/kg + C10	170.60 ± 35.80	1.11 ± 0.23	136.48 ± 56.45	2.08 ± 0.85	3.23 ± 1.24	20 (20/20)
50 IU/kg + C10	515.25 ± 121.80	1.55 ± 0.37	213.69 ± 70.72	1.50 ± 0.51	5.61 ± 2.23	20 (20/40)
75 IU/kg + C10	810.70 ± 239.07^**^	1.72 ± 0.47	1004.81 ± 365.53^***^	4.61 ± 1.66^*^	15.73 ± 4.81^*^	20 (20/40)
75 IU/kg	164.25 ± 63.41	0.33 ± 0.13	37.93 ± 29.66	0.18 ± 0.15	0.56 ± 0.40	40 (20/90)
**Liquid**						
25 IU/kg + C10	484.60 ± 88.429	2.91 ± 0.53^***^	410.06 ± 223.51	5.71 ± 3.11	10.12 ± 5.62	20 (20/40)
50 IU/kg + C10	575.50 ± 297.69	1.73 ± 0.89	495.46 ± 312.03	3.45 ± 2.17	9.49 ± 6.23	20 (20/60)
75 IU/kg + C10	748.60 ± 219.43	1.50 ± 0.44	595.08 ± 349.45	2.76 ± 1.75	14.08 ± 7.29	20 (20/40)
75 IU/kg	358.10 ± 167.64	0.72 ± 0.34	216.04 ± 214.93	1.00 ± 0.99	3.23 ± 4.95	20 (20/360)

### Histomorphological assessment showed similar epithelial erosion and villi morphology after exposure to mini-tablets and liquids containing C10

**Fig. 5:**
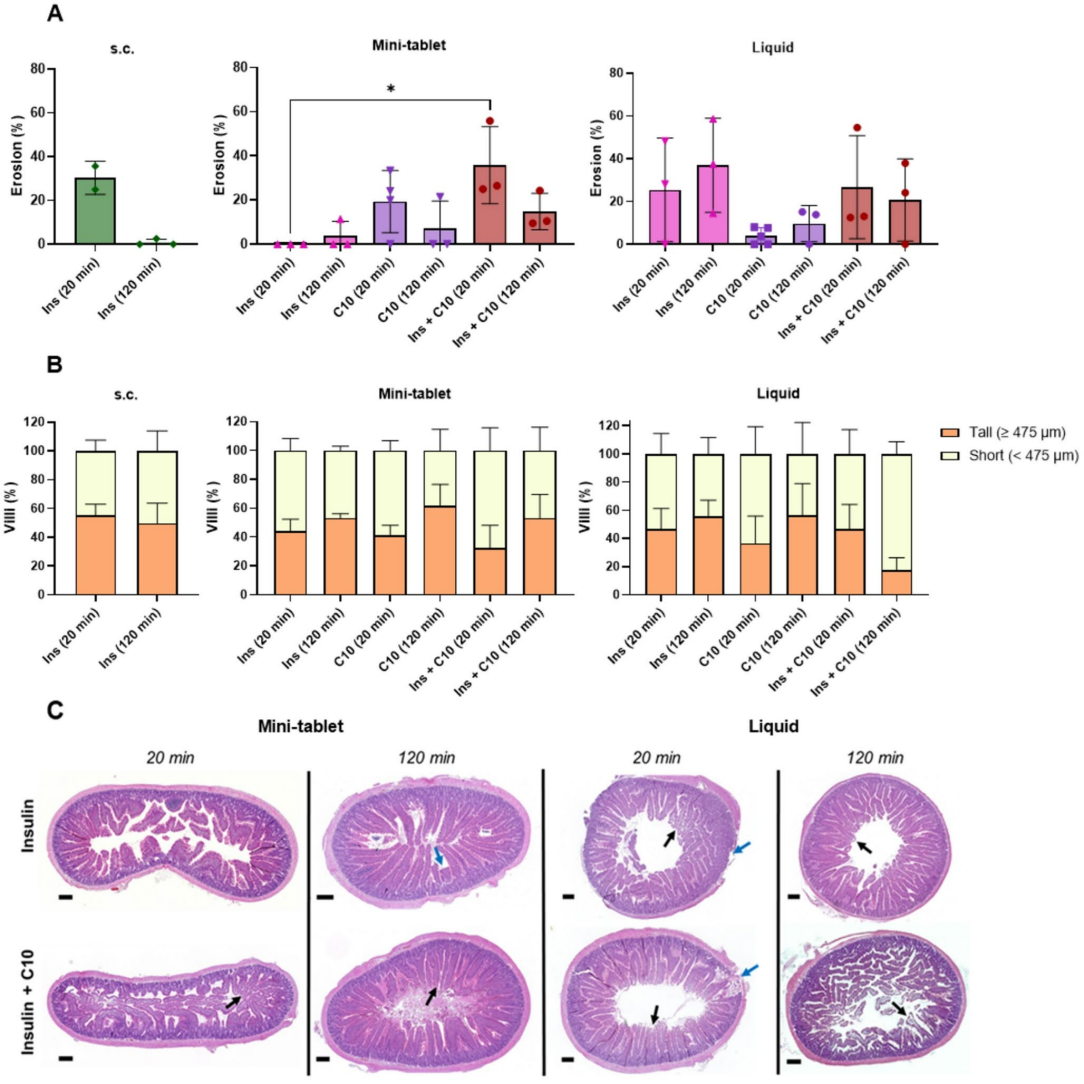
The histomorphological assessment of rat intestinal tissue exposed to tablets or liquids containing insulin (75 IU/kg), C10 (26 mg/kg), or insulin in combination with C10 for 20 and 120 min. Intestinal samples from rats administered s.c. insulin (1 IU/kg) were used as untreated controls. (**A**) The proportion of villi exhibiting epithelial erosion or disruption of the lamina propria, (**B**) the classification of each villus according to its height, and (**C**) representative images of H&E-stained tissue following exposure to insulin, and C10. Brackets indicate statistical differences between groups were *p < 0.05. Arrows indicate areas of erosion (black) and procedural damage (blue). Images were exported at the optical equivalent of 4× magnification. Scale bar = 250 μm. Data was collected for *N* = 2–3 (s.c.), *N* = 3–4 (mini-tablets), and *N* = 3–5 (liquid formulations) (*n* = 1)

To determine the impact of C10 inthe two dosage forms on the intestinal mucosa and to capture mucosal repair, tissue samples were assessed for erosion and morphological changes after 20- and 120-min exposures to mini-tablets and liquids containing C10. Tissue exposed to mini-tablets of C10 (26 mg/kg), and insulin (75 IU/kg) + C10 showed a greater degree of epithelial erosion after 20 min compared to the mini-tablets containing insulin alone (Fig. 5A and C). In particular, the tablets containing insulin and C10 showed a significant increase in erosion at 20 min. However, by 120 min, the proportion of eroded villi was reduced, indicating rapid mucosal repair. The tissue samples exposed to the insulin + C10 liquid formulation followed a similar pattern to the mini-tablets, but did not show any significant erosion (Fig. 5A and C). This was likely due to the unusually high degree of erosion seen in some of the insulin control samples and, indeed, also in the s.c. 20 min samples included as controls. This could thus be procedural damage, as can also be seen from the mini-tablet samples that insulin alone does not cause erosion. Importantly, the fact that the s.c. control at 20 min also displayed detectable erosion different from the 120-min samples, show that assessment of such effects can vary highly between animals and that conclusions based on erosion effects should be done cautiously.

However, when villi morphology was examined in regard to the proportion of “Tall” and “Short” villi, this was the same after 20 and 120 min in the control. Also, the values were approximately the same across all samples (Fig. 5B). Overall, this indicates that neither dosage form of C10 induced structural changes in the intestine beyond erosion, which was largely repaired within 120 min.

## Discussion

The aim of this study was to evaluate whether it is feasible to test a compacted solid dosage form, more specifically mini-tablets, containing the permeation enhancer C10 for oral peptide drug delivery studies in the commonly used rat model. We compared efficacy and compatibility to that observed with the corresponding liquid dosage forms in terms of PK/PD and histomorphology.

While the T_max_ values were overall similar for all the formulations administered intra-intestinally, the C_max_ and AUC values expectedly showed higher amounts of insulin absorbed when increasing concentrations of insulin were co-administered together with the fixed dose of C10. The enhancing effect of C10 in the selected dose agrees with previous studies using liquid formulations resulting in increased intestinal absorption of macromolecules following co-administration with C10 [10, 15–18]. The studies by Berg et al. and Maher et al. showed 13-fold and 33-fold increases in the AUCs, respectively, after intra-intestinal co-administration of fluorescein isothiocyanate dextran 4,000 (FD4) with 100 mM C10 to rats when compared to administration without C10 [15, 16]. Berg et al. administered 100 mM C10 in 0.8 mL (pH 6.5) to rats with an average weight of 285 g resulting in a C10 dose of 56 mg/kg [16], whereas Maher et al. used 100 mM C10 solution with FD4 was administered in 0.2 mL to rats with a weight between 200 and 250 g which resulted in a C10 dose between 15.5 and 19.5 mg/kg [15]. In our study, 26 mg/kg corresponding to 100 mM C10 administered in a liquid form together with insulin at 25–75 IU/kg increased the AUC of insulin in a linear concentration-dependent manner indicating that the insulin concentration dictated absorption and that the effect of C10 was similar irrespective of the insulin dose. In comparison, co-administering the same dose of C10 with 75 IU/kg insulin in the solid dosage form resulted in a 26-fold increase in both AUC and BA, and the difference was significant at specific time points in the PK profile (Fig. 4). However, a linear correlation to the insulin dose was not evident for the mini-tablets showing that the effect of C10 was indeed different for the higher insulin dose. For comparison, doses of 550 mg C10 in tablets were tested in a phase 2 clinical trial for delivery of insulin glargine in subjects of approximately 90 kg with a starting dose of 2700 nmol, corresponding to 16 mg insulin glargine per dose, i.e. the PE: API ratio (w/w) was in this case 34:1 [13] and thus in range with the ratios used in our study. For comparison, the PE: API ratio´s (w/w) used in Rybelsus^®^ tablet incorporating 300 mg SNAC range from approximately 200-6:1 [6].

When comparing the liquid and the solid dosage forms, an overall trend towards higher absorption of insulin when comparing AUCs, and corresponding higher bioavailabilities, was observed for the liquid formulations with the lower doses of insulin (25 IU/kg and 50 IU/kg) and C10 than for the corresponding mini-tablets. However, for the higher dose of 75 IU/kg insulin dosed together with C10, the AUC was significantly higher after dosing the mini-tablets than the AUC obtained for the liquid counterpart (*p* < 0.05). It should be noted that the higher relative increase in BA for the 75 IU/kg mini-tablets with C10 than for the corresponding liquid formulation can be attributed to the difference in the controls without C10, though this was not significant.

Several reasons can explain the observed differences: Primarily, the liquid formulations are not dependent on disintegration and solubilization of the insulin and the C10 offer the advantages of dosing pre-dissolved drug and a (some) dissolved PE, which can lead to a faster onset of action of the C10 interacting with the mucosal membrane to [11, 32]. Although these highly concentrated C10 formulations are dosed as suspensions, the liquid phase is saturated with dissolved C10, and further dissolution will occur upon dosing, subsequent absorption of C10 [16], and mixing with intestinal constituents. However, the distribution of the dosed liquid volume over a larger surface area and mixing with intestinal fluid as a result of the transit through the intestine will also dilute the local concentrations of both C10 and insulin, and when the concentration gradient across the mucosa decreases, this will affect passive diffusion of both insulin and C10 into and across the mucosa according to Fick’s first law of diffusion [33]. On the other hand, the mini-tablets must disintegrate before the insulin and C10 are dissolved and become available for absorption and for exerting enhancing effects, respectively. We observed that mini-tablet disintegration was slower when containing 26% (w/w) of C10 which aligns with previous studies showing that MCFAs exhibit a similar or higher contact angle compared to microcrystalline cellulose and a higher contact angle than lactose monohydrate, which were used as fillers in this study [34–36]. The decrease in filler content and the increase in C10 could therefore hinder fluid penetration (wicking) into the tablet, thereby prolonging the disintegration process in the relatively small intestinal volume. The in vitro disintegration demonstrated that all mini-tablets disintegrated in less than 12 min (Table 3), that in vitro release of insulin showed similar insulin release profiles (Fig. 2) irrespective of the content of insulin suggesting that no relevant insulin-excipient interactions determined the function of the insulin release, and that in vivo disintegration of comparable mini-tablets occurred rapidly (within < 5 min) in the low intestinal volume in vivo (Fig. 2). Although the PK/PD responses to some degree depended on the dose of insulin in the mini-tablets, we cannot conclude on whether synchroneity in the release of insulin and C10 from the mini-tablets, and thereby co-localization of the PE and the insulin at desired concentrations may have influenced the PK/PD results obtained with the mini-tablets. However, as the mini-tablets did not move notably in the intestine, the dosed C10 is assumed to be in proximity with a smaller area of the intestinal mucosa compared to when it is in a liquid form. Thus, resulting in a higher local concentration of C10 and insulin leading to a higher permeation enhancer effect and insulin absorption. Related to our finding that the release of insulin in PBS was not affected by the C10 content (Fig. 2), a very recent study showed somewhat similar release profiles of insulin and C10 from mini-tablets in simulated intestinal buffer with >80% insulin and C10 released within 20 min and 10 min, respectively. They applied a 100 × larger volume, which is likely the reason for the more rapid dissolution compared to our observations [37]. Storage stability of the tablets for up to four weeks is not expected to influence these outcomes as: No visual difference in the appearance of the tablets (such as capping or discolouring) was observed, no occurrence of extra peaks in the HPLC chromatograms used to quantify insulin release were observed, and the total amount of released insulin in vitro was in no case different from the incorporated amount (represented in Fig. 2 as 100%) for each of the three doses of insulin. Adding to this, all tablets including the PE displayed some effect on blood glucose in vivo (Fig. 4; Table 5) supporting that the activity of the insulin was not compromised during processing and storage.

There are several publications reporting on absorption of insulin in rats after dosing in combination with different PEs or by other formulation strategies to improve transmucosal permeation, but most of these are employing liquid formulations [19]. Nevertheless, oral administration in vivo in rats has also been done with flat faced tablets mainly consisting of compacted insulin and functional excipients such as thiolated chitosan (diameter 1.5 and 5 mm) [38, 39], thiomer nanoparticles compacted in a tablet (diameter 2.5 mm) [40], and chitosan-aprotinin tablets (diameter not reported) [41]. However, very few studies in rats report on the effect of tablets containing several excipients with different functionalities – such as filler, disintegrant, binder, and lubricant together with a PE – resembling a final dosage form for human use. One study used compacted mini-tablets (diameter 2.5 mm, curvature radius 3 mm, height 2.2 mm) with insulin, sodium glycocholate (PE), sodium starch (disintegrant), magnesium stearate (lubricant), and microcrystalline cellulose (filler), which were coated for colonic delivery after oral gavage in rats [42]. To our knowledge, the current study is the first reporting administration of tablets containing insulin and the PE C10 with excipients such as crospovidone (disintegrant), copovidone (binder), magnesium stearate (lubricant), microcrystalline cellulose and monohydrate lactose (fillers) to rats. The mini-tablets produced and tested here contain excipients, which may conceivably be included in a final tablet for human use, with tensile strengths sufficient to withstand commercial manufacture and handling [29]. Both for the mini-tablets and the liquid formulations, the study displays relatively high variance in the PK/PD data. This is in part explained by the fact that some rats did not respond to the formulations. For the liquid formulations, the high variance could be due to dilution and distribution in a larger area and thus variable concentrations of PE and API at the site of absorption compared to the mini-tablets, which more or less remained at the site of administration. On the other hand, for the mini-tablets, a contributing factor to the variance could likely be differences in the intestinal liquid volume at the site of administration since the total volume is only ~ 1.9 mL throughout the small intestine of the rat [27]. Limited volumes might be sufficient to disintegrate the mini-tablets but not be sufficient to dissolve the entire content of the mini-tablet, resulting in a higher degree of variance in the absorption. Fagan et al. reported the equilibrium solubility of C10 in 1 mL of intestinal buffer to be >250 mg/mL supporting that solubility of the dosed 7.8 mg (~ 35.5% w/w) C10 in the intestinal lumen would be possible if some liquid was present [37]. Overall, this study contributes with knowledge regarding the use of the rat model for evaluating absorption from the small intestine after dosing mini-tablets instead of simple liquid formulations. To which extent the effect of solid dosage forms can be translated and how the anatomical differences to human will limit the translation of data cannot be speculated at this stage.

While the use of PEs in general may raise concerns in relation to intestinal mucosal damage and associated extensive disruption of intestinal integrity thereby risking absorption of pathogens, to date the use of C10 as a PE has not given rise to any serious adverse effects [10, 43–45]. Moreover, C10 was included in the approved rectal suppository with ampicillin (Doktacillin^®^, Meda, Solna, Sweden) [46]. Additionally, C10 is approved by the FDA as a food additive with the World Health Organization not imposing any limit on daily intake [41]. In our study, histomorphological assessment of C10 exposed tissue showed repair of erosion after 120 min, which agrees with other studies. For example, a study by Berg et al. demonstrated full recovery of the rat intestinal epithelium 60 min after intestinal administration of C10 solutions (50–100 mM) indicating a reversible effect on the epithelium after C10 exposure [16]. Overall, our findings did not show differences between dosing mini-tablets or liquids containing C10 and indicated that villi height together with erosion can paint a more complete picture of the effect on the mucosal wall.

## Conclusion

This work demonstrates that it is possible to achieve a decrease in blood glucose and increase in insulin plasma levels when administering mini-tablets as well as liquids with insulin in combination with C10. However, PK/PD readouts may be different when administering mini-tablets intestinally compared to liquid formulations such as suspensions, despite the formulations containing the same amounts of insulin and a fixed amount of the PE C10. A significantly higher response was seen for the mini-tablet with 75 IU/kg insulin and C10 compared to the suspension with 75 IU/kg and C10, which is likely a result of the high local concentration of both C10 and insulin at the absorption site. Both dosage forms containing C10 seemed to cause epithelial erosion within 20 min of intestinal exposure, but mucosal repair was evident after 120 min, indicating that C10 is well tolerated both when dosed in mini-tablets and as liquids. Overall, the findings in this study suggest that mini-tablets can be used to assess peptide bioavailability and the effect of PEs in the rat as a preclinical model.

## Supplementary Information

Below is the link to the electronic supplementary material.

Supplementary Material 1

## Data Availability

The datasets generated during and/or analysed during the current study are available from the corresponding author on reasonable request.
