# Supplementary material for: Impact of sodium caprate dosed as a mini-tablet or suspension on insulin delivery and mucosa histomorphology
Source: Drug Deliv Transl Res. 2025 Oct 8;16(6):1773–87. doi: 10.1007/s13346-025-01977-8 (PMC13183742; doi:10.1007/s13346-025-01977-8)
Supplement: Supplementary file 1 — Supplementary Material 1 [file 13346_2025_1977_MOESM1_ESM.docx]

# Supporting information

Impact of sodium caprate dosed as a mini-tablet or suspension on insulin delivery and mucosa histomorphology

*Freja Fredholt, Joanne Heade, Jukka Rantanen, Stine Rønholt, Hanne Mørck Nielsen*

## K-means clustering of villi height measurements taken from s.c. control intestinal samples


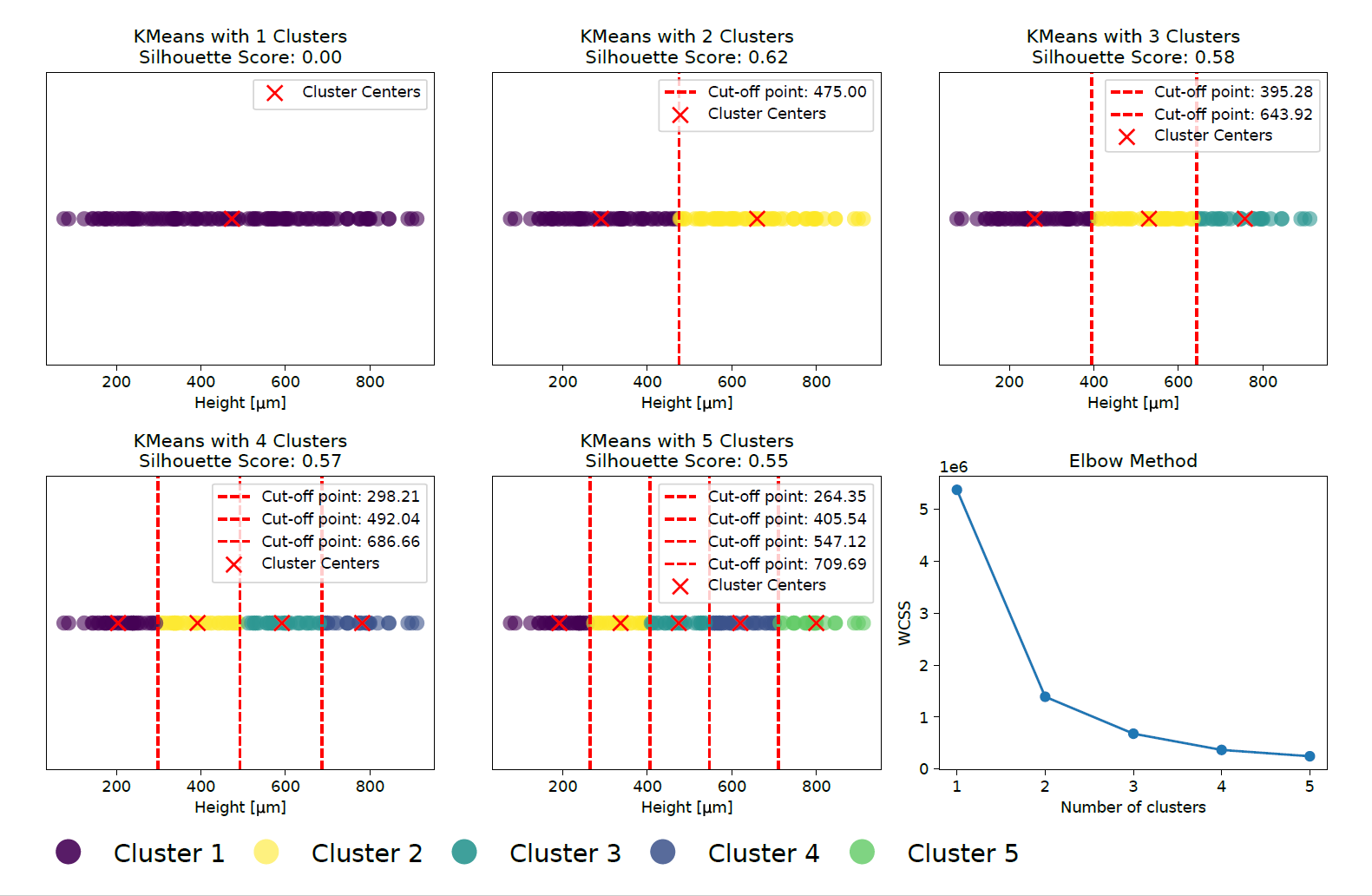


**Figure S1:** K-means clustering of villi height data from untreated intestine harvested from the s.c. control animals (harvested at 120 min), showing the cut-off value between clusters. The Elbow Method helps determine the optimal number of clusters, while the silhouette score indicates the quality of the clusters from -1 (poor clustering) to 1 (good clustering). Two clusters were found to be optimal (silhouette score = 0.62) and the cut-off point was 475 µm.
